# Solvent and cosolute dependence of Mg surface enrichment in submicron aerosol particles†

**DOI:** 10.1039/d1cp04953d

**Published:** 2022-01-14

**Authors:** Eetu Pelimanni, Clara-Magdalena Saak, Georgia Michailoudi, Nønne Prisle, Marko Huttula, Minna Patanen

**Affiliations:** Nano and Molecular Systems Research Unit, Faculty of Science, University of Oulu Box 3000 90014 Finland eetu.pelimanni@oulu.fi minna.patanen@oulu.fi; Department of Physics and Astronomy, Uppsala University Box 516 75120 Uppsala Sweden; University of Vienna, Department of Physical Chemistry Währinger Straße 42 1090 Vienna Austria; Center for Atmospheric Research, Faculty of Information Technology and Electrical Engineering, University of Oulu P. O. Box 4500 90014 Finland

## Abstract

The formation of multicomponent aerosol particles from precursor solution droplets often involves segregation and surface enrichment of the different solutes, resulting in non-homogeneous particle structures and diverse morphologies. In particular, these effects can have a significant influence on the chemical composition of the particle–vapor interface. In this work, we investigate the bulk/surface partitioning of inorganic ions, Na^+^, Mg^2 +^, Ca^2 +^, Cl^−^ and Br^−^, in atomiser-generated submicron aerosols using synchrotron radiation based X-ray photoelectron spectroscopy (XPS). Specifically, the chemical compositions of the outermost few nm thick surface layers of non-supported MgCl_2_/CaCl_2_ and NaBr/MgBr_2_ particles are determined. It is found that in MgCl_2_/CaCl_2_ particles, the relative abundance of the two species in the particle surface correlates well with their mixing ratio in the parent aqueous solution. In stark contrast, extreme surface enrichment of Mg^2 +^ is observed in NaBr/MgBr_2_ particles formed from both aqueous and organic solution droplets, indicative of core–shell structures. Structural properties and hydration state of the particles are discussed.

## Abstract 2

*In situ* XPS of aerosol particles formed from aqueous and EtOH solutions of Mg and Na bromides show surface enrichment of Mg, while particles grown from aqueous solution of Ca and Mg chlorides do not exhibit surface segregation.

## Graphical abstract

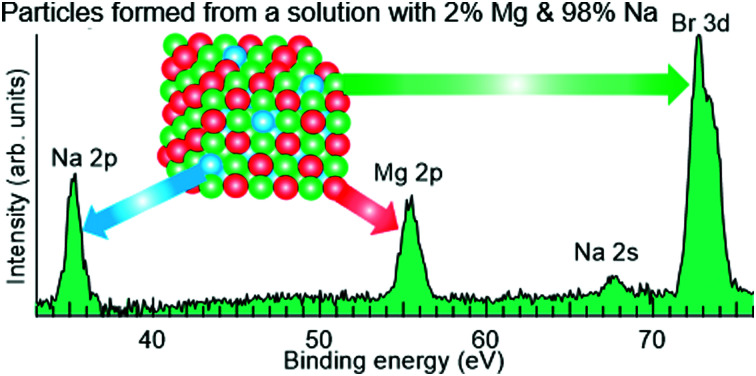

## 1 Introduction

The internal structure of aerosol particles formed from precursor solution droplets, *e.g.* in industrial spray-drying processes^1^ or natural sea spray,^2^ is often non-uniform. As the solvent is gradually evaporated and concentration increases in the finite-sized droplet environment, the different chemical species can segregate into sub-units in adjacent or core–shell configurations.^3–7^ As a consequence, certain species, possibly with seemingly negligible concentrations in the parent solution, can become enriched in the particle surface. Such processes should be accounted for when the particle surface properties and thereby the chemical reactivity are considered, yet they remain incompletely understood.

In recent years, surface sensitive and chemically specific X-ray spectroscopic techniques have been applied at high brilliance synchrotron radiation facilities for determining the surface properties of submicron particles. Especially atmospherically relevant particles have been probed with X-ray photoelectron spectroscopy (XPS) and X-ray absorption spectroscopy (XAS), as freestanding when generated *in situ* using atomisers in combination with aerodynamic lens inlets, and as deposited on substrates. A variety of different structural phenomena in freestanding salt nanoparticles has been uncovered in works by different groups: Antonsson *et al.*^4^ observed surface enrichment of Br in freestanding NaBr/NaCl particles. In NaCl/Na_2_SO_4_ particles, Antonsson *et al.*^6^ found that the inability of the species to co-crystallise resulted in surface enhancement of the minority species. Unger *et al.*^7^ observed core–shell type structures in artificial sea spray aerosol (SSA) particles, discussing also that substrate deposition may affect the particle structure, specifically the formation of core–shell structures *vs.* adjacent crystal moieties (full *vs.* partial surface coverage, respectively). Kostko *et al.*^8^ and Abid *et al.*^9^ have utilised XAS to study the hydration state of freestanding inorganic and mixed organic/inorganic nanoparticles. Lin *et al.*^10^ recently probed pre-deliquescent water uptake in deposited NaCl, sucrose and malonic acid particles.

In continuation of these studies, here we determine the chemical composition of the outermost few nm thick surface layer of freestanding submicron particles atomised from aqueous binary salt mixtures of MgCl_2_/CaCl_2_ and NaBr/MgBr_2_. Specifically, the relative ion concentrations and water content in the particle surface are determined. Additionally, the role of the solvent in defining the particle surface composition is assessed by atomising the Br-salts also using an organic solvent (ethanol), and comparing the results to the aqueous case.

## 2 Experimental

### 2.1 Overview

Surface enrichment was studied by atomising solutions with varied salt mixing ratios, and comparing the observed relative salt concentrations in the particle surface to those in the parent solution. The experiment was carried out at the soft X-ray beamline PLÉIADES at Synchrotron SOLEIL.^11^ Aerosol particles were generated from room temperature liquid solutions with a constant output atomiser (TSI 3076), using nitrogen (2 ± 0.5 Bar) as a carrier gas. The solutions were prepared using ultrapure water (Milli-Q) or ethanol (≥99.8%, Sigma Aldrich) as a solvent, to which powder form MgCl_2_ (anhydrous, ≥98%, Sigma), CaCl_2_·2H_2_O (≥99%, Alfa Aesar), NaBr (anhydrous, ≥99%, Sigma Aldrich) and MgBr_2_·6H_2_O (99%, Aldrich) were added with varying mixing ratios (details given in ESI†). The total salt concentration was <6g L^−1^ (<50 mmol L^−1^) in all samples. Before their characterisation, the particles generated from aqueous solutions were passed through a two-stage diffusion dryer (TSI 3062) section to decrease the ambient relative humidity level (RH). After the drying section, the constant particle flow was divided to the main XPS experiment and a separate granulometry analysis. For the ethanol solutions, the drier section was replaced by mildly heated (∼45 °C) tubing along with a cold trap.

### 2.2 XPS analysis

For the XPS analysis, the particles were introduced into a vacuum chamber using the “Multi-Purpose Source Chamber” (MPSC) of the PLÉIADES beamline, in which a focused particle beam was produced by an aerodynamic lens and a beam skimmer.^12^ The particle beam was irradiated with soft X-ray synchrotron radiation (SR), and a VG-Scienta R4000 hemispherical deflection analyser with a position sensitive detector was used to record the photoelectron spectra. The elemental surface composition of particles was determined from relative intensities of the recorded photoelectron peaks, which are proportional to the relative number concentrations of the elements. Due to the high inelastic scattering probability of electrons traveling through the particle, the main photolines are comprised of electrons of which >90% originate from a depth of only ≲2.5 nm (assuming typical inelastic mean free paths of inorganic compounds^13^). As the spectra are collected from a constantly renewed beam of non-supported and randomly oriented particles, the average particle surface composition is effectively determined. The results are also not subject to any potential substrate induced effects or permanent radiation damage.

The particle beam, the X-ray beam and the analyser were installed perpendicular with respect to each other. Linearly polarised light was used with the polarisation axis in the so called “magic angle” of 54.7° with respect to the observation axis, minimising angular dependency of the relative photoelectron yields. The electron analyser was operated with a pass energy of 100 eV and a 0.8 mm entrance slit size was used. Binding energies (BEs) are given with respect to the vacuum level, calibrated to the outermost valence peak (X-state) of molecular N_2_ at 15.60 eV.^14^ Based on the observed Full Width at Half Maximum (FWHM) of the same peak, the overall energy resolution is estimated to be ∼300 meV in the measurements conducted with a photon energy of 175 eV. Peak analysis was performed using the Igor Pro software by WaveMetrics, with a least-squares curve fitting package SPANCF.^15,16^

### 2.3 Particle size analysis

**Fig. 1:**
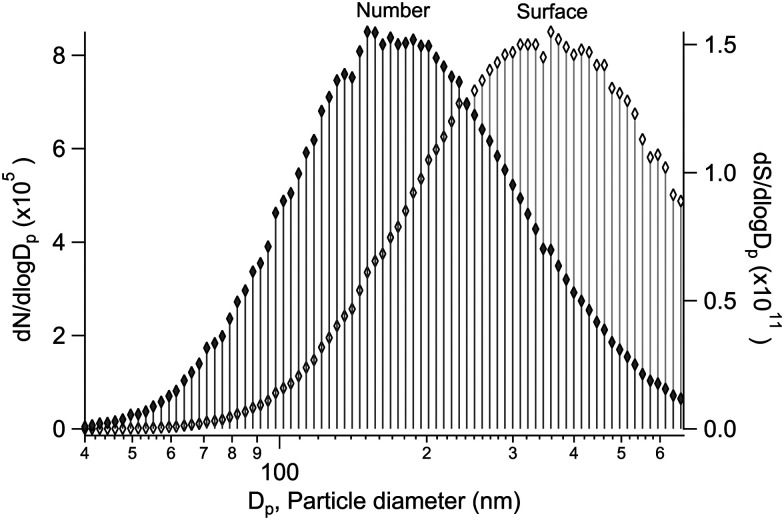
A typical diffusion loss corrected particle size distribution produced, as observed in the SMPS after the drier section. Both the normalised number (*N*) and surface (*S*) concentration distributions (with spherical particle assumption) are shown.

Alongside the main XPS experiment, a separate granulometry analysis was carried out. The particle size distribution was monitored using a scanning mobility particle sizer (SMPS), which included an electrostatic classifier (TSI 3080) and a differential mobility analyser (DMA) (TSI 3081), a soft X-ray aerosol neutraliser (TSI 3087) and a condensation particle counter (CPC) (TSI 3786). In the majority of the samples, the geometric mean particle size was between ∼100–200 nm, with a geometric standard deviation of ∼1.5–1.8 (see ESI†). As an example, a typical size distribution is shown in Fig. 1, obtained for particles generated from a mixed aqueous solution of MgBr_2_·6H_2_O/NaBr with a 50/50 mol% solute mixing ratio. As the size distribution is further subject to the transmission function of the aerodynamic lens before the XPS analysis, we note that a transmission efficiency close to unity is expected for the lens in the given mean size regime and qualitatively the effect should be a reduction of much smaller and much larger particles.^12,17,18^ Also, as the ionisation probability of a particle is proportional to its size (surface area), the results effectively reflect a somewhat larger size distribution than actually present in the beam. Overall, the particles are thus categorised as “submicron”.

## 3 Results & discussion

### 3.1 Data analysis

**Fig. 2:**
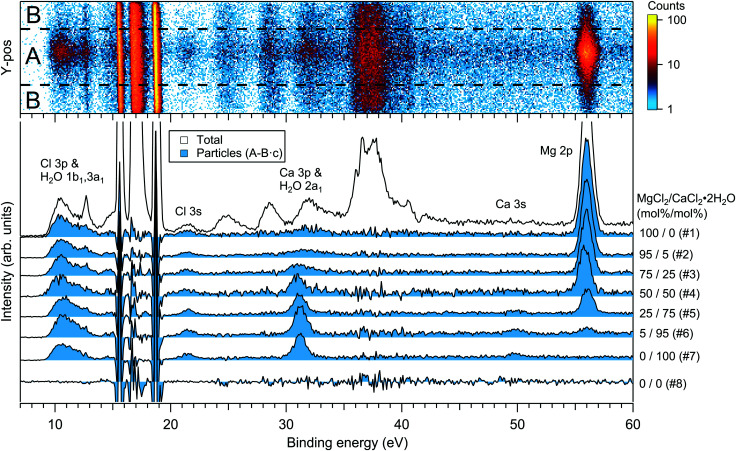
Photoelectron spectra of MgCl_2_/CaCl_2_ (H_2_O) particles. The 2D-map on top shows the detector *Y*-position *vs.* electron binding energy for spectrum #1. The photon energy was 175 eV.

**Fig. 3:**
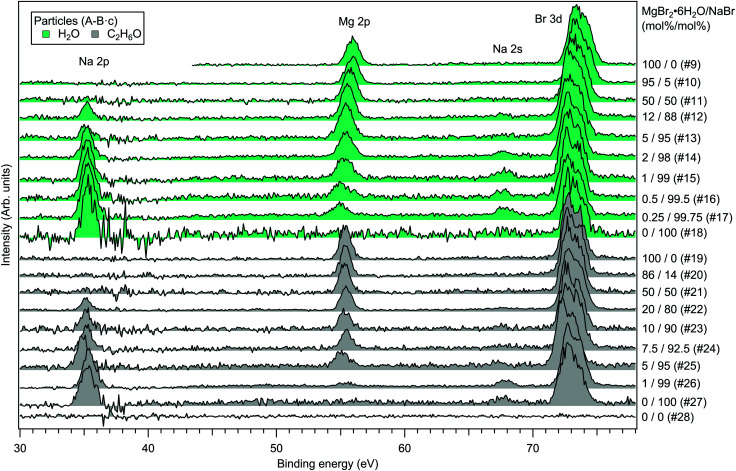
Photoelectron spectra of MgBr_2_/NaBr (H_2_O) and MgBr_2_/NaBr (C_2_H_6_O) particles. The photon energy was 175 eV.

For the particles grown from aqueous solutions of MgCl_2_ and CaCl_2_, from now on marked as MgCl_2_/CaCl_2_ (H_2_O), relative abundances of the two salts at the particle surface were determined from intensities of the recorded Mg 2p, Ca 3p and Cl 3s signals. The statistics were collected by repeatedly scanning over all three energy levels at a photon energy of 175 eV, thus minimising the effect of particle beam fluctuations and avoiding the need for photon flux normalisation. The spectra are shown in Fig. 2, where the salt mixing ratio and an identifier number is designated for each spectrum. Similar measurements were carried out for the MgBr_2_/NaBr (H_2_O) and MgBr_2_/NaBr (C_2_H_6_O) particles, for which the Na 2p, Mg 2p and Br 3d signals were recorded. Their respective spectra are shown in Fig. 3.

The particle signal is partially overlapped by photoelectron peaks from free H_2_O and N_2_ molecules. The particle and free molecule contributions to the recorded spectra can however be distinguished by taking advantage of their differing spatial distributions in the interaction region. The electron analyser is spatially dispersive in the direction perpendicular to the particle beam propagation axis, here denoted *Y*-axis (with *X*-axis being energy dispersive), so that an electron's position at the detector depends on its point of origin in the interaction region. Electrons coming from the focused narrow particle beam are weighted near the center of the detector, while electrons from the diffuse background gas are spread more evenly (see also ref. 19 and 20). This is visible in the 2D-map shown on the top panel of Fig. 2. A particle-only contribution is obtained by constructing a spectrum from the particle-rich center-region *A*, and subtracting from it a spectrum from the particle-poor leftover region *B*. More specifically, the particle-only spectrum is *A* − *B*·*c*, where *c* is a constant accounting for the slightly different transmission in the two regions (*c* is obtained from the intensity ratio of *A* and *B* in a particle-free reference spectrum #8, measured with only pure water in the atomiser). The remaining background in the particle spectra after subtraction of the molecular contributions is attributed to inelastically scattered electrons originating from the particles.

The particle surface composition is determined from the XPS data by applying two data treatment methods previously used in the literature (see *e.g.* ref. 4 and 6). The use of both methods is motivated by their complementary nature, but also to provide discussion on their accuracy. In “method 1”, atomic percentages (at%) of the different ions within the probed surface layer are determined from the photoelectron peak intensities (*I*), obtained as the fitted peak areas (*A*) corrected with the photoionisation cross sections^21,22^ (*σ*) and kinetic energy dependent transmission of the electron analyser (*T*). For example, the intensity of Mg in the surface of MgCl_2_/CaCl_2_ (H_2_O) particles is *I*_Mg_ = *A*_Mg2p_*σ*_Mg2p_^−1^*T*_Mg2p_^−1^, and at%(Mg) = 100·*I*_Mg_/(*I*_Mg_ + *I*_Ca_ + *I*_Cl_). In “method 2”, the atomic percentages are instead determined by normalising the area ratios (*e.g. A*_Ca3p_/*A*_Cl3s_ and *A*_Mg2p_/*A*_Cl3s_) observed in the mixed particles to those in the single-component reference spectra (*A*_Ca3p_/*A*_Cl3s_ = 4.5 in pure CaCl_2_ particles and *A*_Mg2p_/*A*_Cl3s_ = 18.7 in pure MgCl_2_ particles). In converting these to at%s, it is assumed that Mg^2 +^ and Ca^2 +^ ions are always paired with 2 Cl^−^ or 2 Br^−^ anions, and Na^+^ with 1 anion. Details of method 2 are presented in ESI.†

### 3.2 Results

**Fig. 4:**
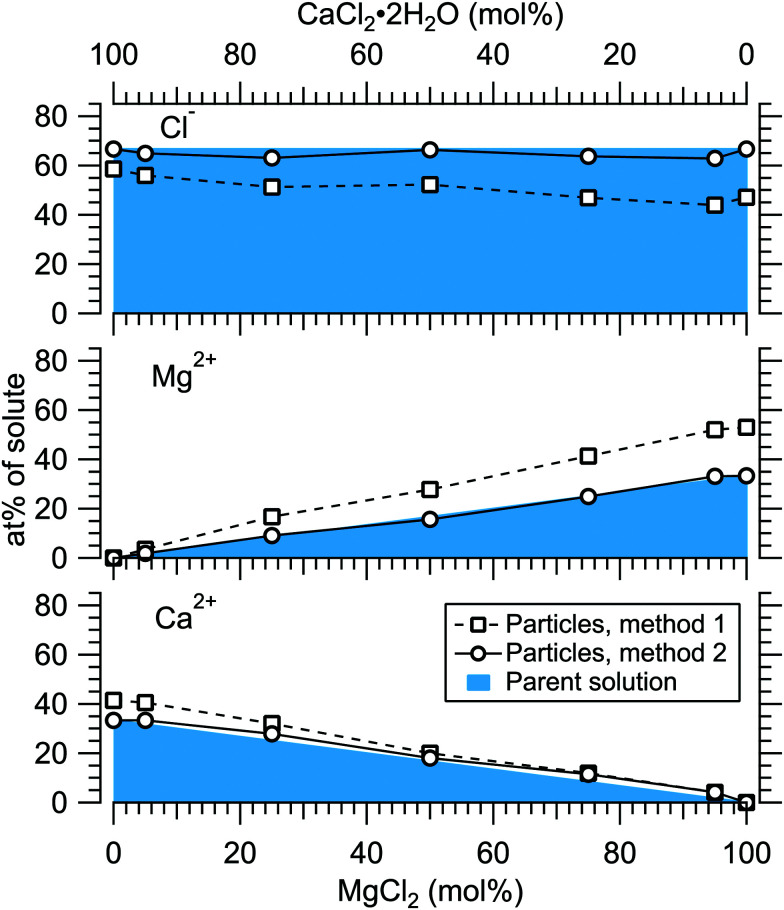
Relative at% of Ca^2 +^, Mg^2 +^ and Cl^−^ at the surface of MgCl_2_/CaCl_2_ (H_2_O) particles plotted as a function of the parent solution composition. The results are shown using two separate data treatment methods. The data corresponds to spectra #1–7.

**Fig. 5:**
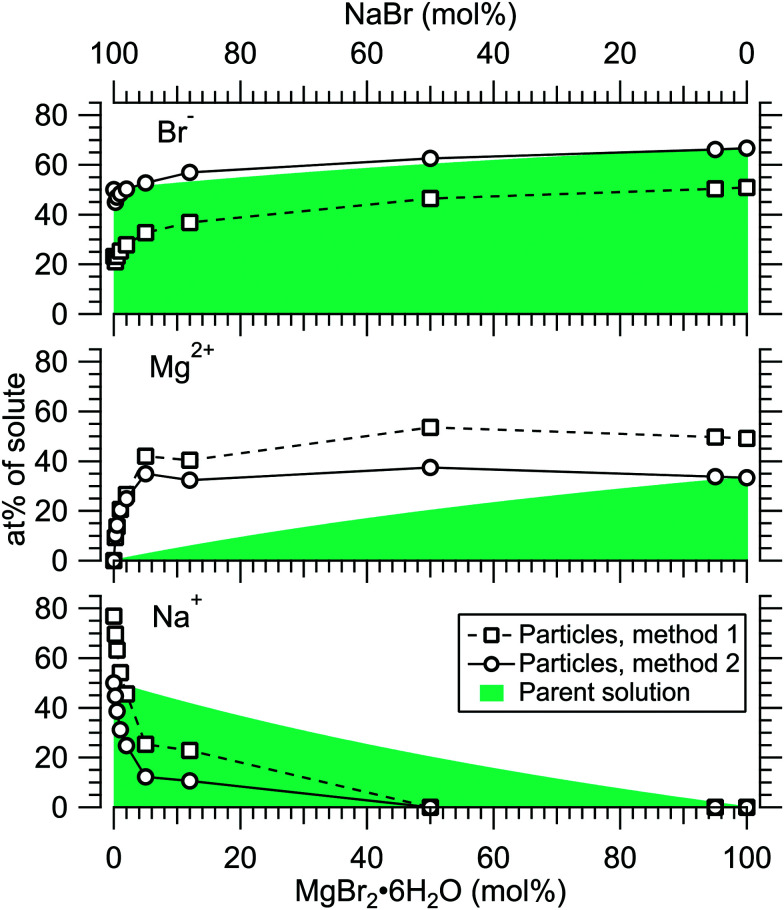
Relative at% of Na^+^, Mg^2 +^ and Br^−^ at the surface of MgBr_2_/NaBr (H_2_O) particles plotted as a function of the parent solution composition. The data corresponds to spectra #9–18.

**Fig. 6:**
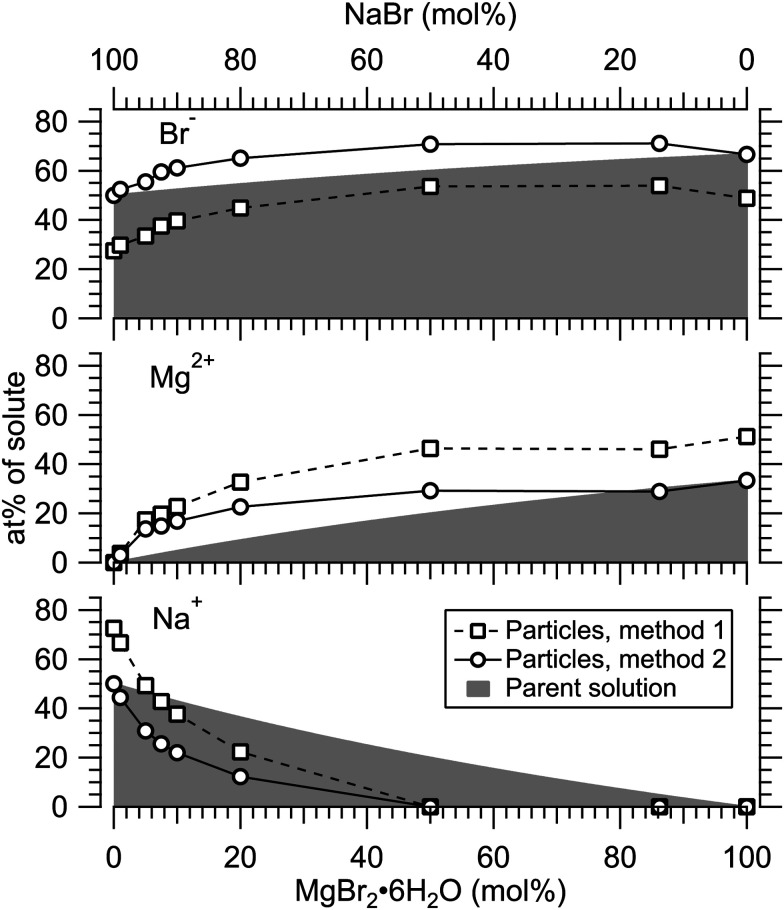
Relative at% of Na, Mg and Br at the surface of MgBr_2_/NaBr (C_2_*H*_6_O) particles plotted as a function of the parent solution composition. The data corresponds to spectra #19–27.

Fig. 4 presents the atomic percentages of Ca^2 +^, Mg^2 +^ and Cl^−^ at the particle surface *vs.* the parent solution composition for MgCl_2_/CaCl_2_ (H_2_O) particles, determined using both methods 1 and 2. Each series of points at a given position on the horizontal axis corresponds to one photoelectron spectrum. Similarly, the at%s of Na^+^, Mg^2 +^ and Br^−^ in the MgBr_2_/NaBr (H_2_O) and MgBr_2_/NaBr (C_2_H_6_O) particles are shown in Fig. 5 and 6, respectively.

It is evident from Fig. 4 that no significant surface enrichment of either Mg^2 +^ or Ca^2 +^ occurs in the MgCl_2_/CaCl_2_ (H_2_O) particles. The measured at%s of Mg^2 +^, Ca^2 +^ and Cl^−^ at the particle surface are seen to correlate well with those in the parent solution, especially in using method 2. Starkly contrasting results are however obtained for the MgBr_2_/NaBr particles. In Fig. 5 and 6, it is seen that Mg^2 +^ is strongly enriched at the particle surface at the expense of Na^+^. In both solvents, with a 50/50 mol% mixing ratio of MgBr_2_/NaBr in the parent solution, the amount of Na^+^ at the particle surface is still practically negligible. Comparing Fig. 5 and 6, enrichment of Mg^2 +^ appears slightly more pronounced in the aqueous case. Even with just 0.25 mol% of the solute being MgBr_2_ (#17), a strong Mg^2 +^ signal is still observed (Mg^2 +^/Na^+^ = 1/400 in the parent solution and 1/5 in the particle surface).

At most, the at%s differ by about 20 percentage points between the two methods, and both methods agree qualitatively indicating no or little segregation in MgCl_2_/CaCl_2_ (H_2_O) particles and significant Mg^2+^ enrichment in the two other systems. Overall, initial assumptions related to both methods are the biggest source of uncertainty in exact quantification of the degree of enrichment, while error bars from statistical uncertainties related to our experimental data should be much less significant. Assuming Poisson statistics, the uncertainty in XPS signal (peak areas) result in ∼1 percentage point variation in at% values. Due to the fact that method 2 is normalised to give the expected bulk stoichiometric ratio of single-component salt particles, it agrees better with the parent solution stoichiometry than method 1. The benefit of method 1 is however that no assumptions for the surface composition are made. While method 2 is more straightforward in that it requires no accurate knowledge of *σ*, spectrometer transmission function, or other contributions, it relies on the assumption that the mixed particles contain structural sub-units similar to those in the single-component salt particles used as references. Deviations from the parent solution composition can thus reflect surface enrichment in the particles, but also *e.g.* non-stoichiometry in cation–anion pairs or changes in hydrate-formation. While here we probed chemically relatively simple particles with only two species and normalisation is therefore straightforward, method 2 especially will be less justified for particles of higher complexity, such as natural SSA where a given cation can exist as a halide, sulfate or carbonate, for example.

For all investigated particles, the anion-to-cation relative signal determined using method 1 (Fig. 4–6) is smaller than expected from stoichiometric arguments and method 2. Because the probe depth covers just a few atomic layers, differences in the number density depth profiles of the cations *vs.* anions can be reflected in the results. In this case, the result could be interpreted as enrichment of cations at the gas-particle interface, which would be rather interesting in light of that opposite behavior (surface enrichment of anions) has been observed in aqueous solutions.^23^ The determined cation/anion balance in the single-component 100% NaBr and 100% MgBr_2_ particles (which are not influenced by the mixing state of different salts) however differ by <5 percentage points between particles grown from aqueous and ethanol solutions, despite of differences in the amount and type of solvent at the surface of the particles. This points to other contributing factors, such as deviation of the effective ionisation cross sections in the particle environment from the atomic values used in the analysis, which are especially prone to variations when the photon energy is not far above the ionisation thresholds.^24,25^ Still, there is strong motivation for using relatively low photon energies to obtain maximal surface sensitivity and signal intensity (strongest ionisation cross sections and also the highest photon flux of dedicated soft X-ray beamlines), and these type of experiments would benefit from more accurate determination of photoionisation cross-sections. We note that if instead of the cross-section and transmission corrected area of the Na 2p peak the corresponding Na 2s peak area is used in the analysis, the Na fraction decreases on average by ∼15 percentage points with an according increase of Br and Mg. Also, from an additionally measured C 1s spectrum (#37) for the 100% MgBr_2_ (C_2_H_6_O) sample, the determined O/C ratio is ∼1/3, while 1/2 would be expected. Ultimately, all the essentials of a photoionisation event in a particle environment should however be grasped, taking into account varying inelastic and elastic scattering cross-sections across the particle surface layers. This leads to iterative modelling of the salt particle surface, where the surface composition is altered until the modelled photoionisation signal matches the observed one.

### 3.3 Particle generation process and hydration state

**Fig. 7:**
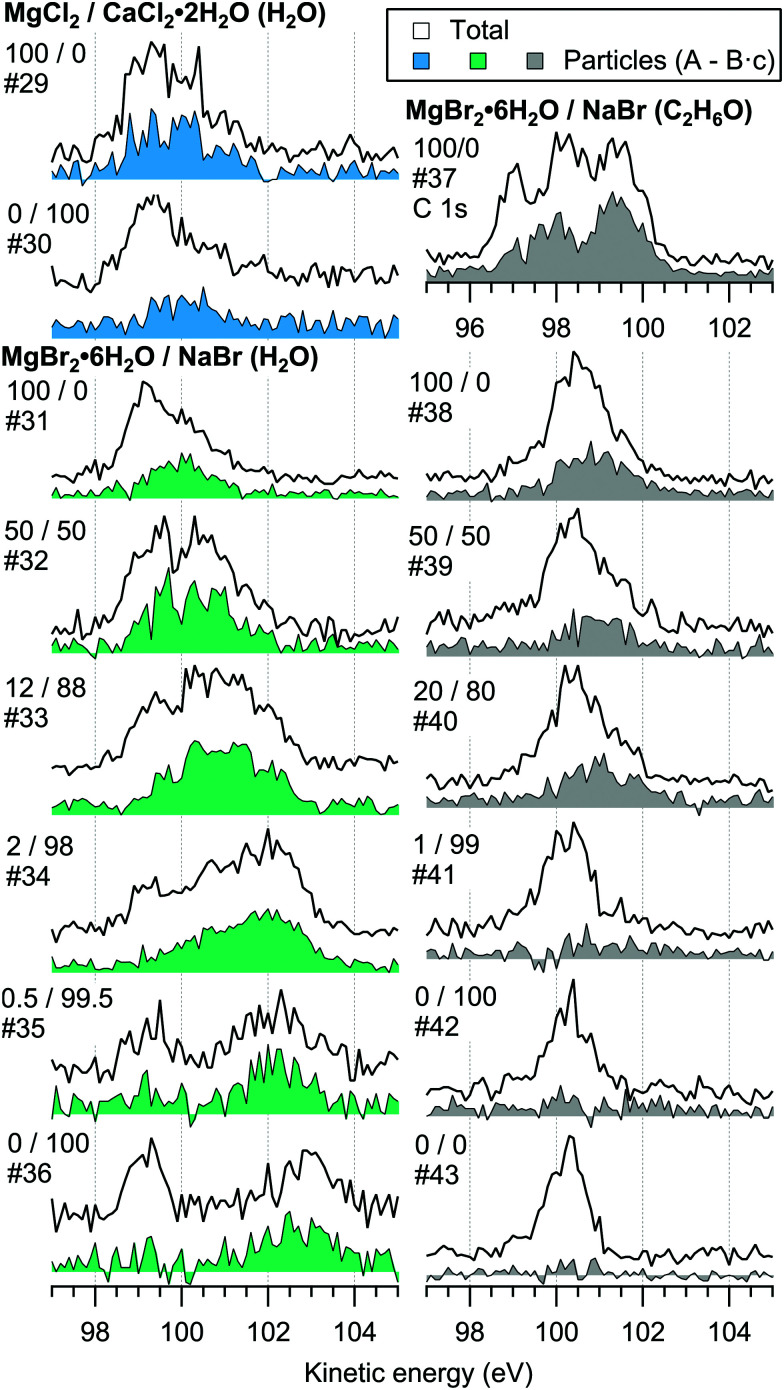
Photoelectron spectra in the O 1s region measured with 640 eV photons. An additional C 1s spectrum (#37) measured with 390 eV photons is also shown.

The retainment of solvent molecules in the particles after passing through the drying stage, the aerodynamic lens and the free flight in vacuum, becomes apparent from the recorded O 1s (and C 1s) spectra, shown in Fig. 7. Also the 1b_1_, 3a_1_ and 2a_1_ valence signatures^26^ from particle water are observed in the low BE region (Fig. 2). The outermost valence feature at ∼11 eV BE likely contains also Cl 3p contribution, based on that the ionisation cross section of Cl 3p is roughly ∼3 times that of Cl 3s.^21,22^ Considering the amount of solvent in the particles, the drying efficiency is reflected to that no signal from condensed solvent molecules is observed when only pure water or ethanol is atomised (#8 and #28). This implies that pure solution droplets do not survive to the interaction region. The amount of solvent in the particles is accordingly low relative to salt: the measured O/Mg atomic ratio (within the probe depth in the particle surface) is ∼5/1 for 50/50 mol% MgBr_2_/NaBr (H_2_O) and ∼2/1 for 100 mol% MgCl_2_ (H_2_O) particles, while the O/Ca ratio is ∼1/2 for 100 mol% CaCl_2_ (H_2_O) particles. These were determined from the O 1s/Mg 2p and O 1s/Ca 3p intensity ratios. Since these peaks (as well as the O/C ratio from O 1s and C 1s mentioned above) were measured separately with different photon energies, the intensities were corrected by the measured photons per s (from a current of AXUV100 photodiode corrected for varying responsivity between used energies), the number of recorded scans and photoionisation cross sections. In the ethanol case, a stronger O 1s signal is obtained from the particles when MgBr_2_·6H_2_O is included in the solution, compared to the pure NaBr sample (#42). Note that the amount of solvent in the particles is not considered in Fig. 4–6, but only the relative abundance of the ions.

The hydration state of the particles depends on ambient conditions, most importantly of RH which governs the evaporation dynamics. In our experiment the particles are probed in high vacuum, but it is appropriate to discuss the preceding conditions in some more detail. The RH after the ∼1.5 m long silica drier section was measured to be in the <40% regime, near the lens entrance. This is above the efflorescence points of some of the investigated salts, but nevertheless only a relatively small amount of water is observed in all particles as discussed above. It is likely that significant solvent evaporation occurs also downstream in the lens section.^27,28^ The particles enter the lens through a flow limiting pinhole <1 mm in diameter, ∼0.3 mm in our experiment, after which the pressure drops to a few mbar and RH is significantly lowered. The few mbar pressure is maintained all the way to the exit of the lens,^18^ after which a few cm of free flight follows at ∼10^−3^ mbar before and ∼10^−6^ mbar after the beam skimmer, until the particles are finally probed with X-rays. Evaporation has a cooling effect which in the lens section is compensated by heat input from the surrounding gas (if continued in the following high vacuum region the particle temperature can be significantly lowered).^27–29^ Zelenyuk *et al.*^30^ have observed that hygroscopic droplets (generated using the same atomiser model as in our experiment) lose a significant fraction of the water during their few millisecond long transit time through an aerodynamic lens. They concluded that effort should nevertheless be put in pre-drying the particles, as for example the evaporation dynamics of NaCl particles in the lens were found to be somewhat complex. Based on the ≲0.8 L min^−1^ flow rate and volume of the lens section we estimate that in our experiment the gas residence time (and thereby the particle transit/desiccation time) is considerable,^27^ on the order of some hundreds of milliseconds. The MPSC design includes a noteworthy ∼1.5 m travel section in between the entrance pinhole and the actual ∼ 0.5 m long lens component, which increases the transit time and evaporation efficiency.^12^

The presence of water in the particles after the drying cycle is not surprising in light of that even after efflorescence, hydrated forms are expected for all the salts studied here. In this case, potential candidates include CaCl_2_·*X*H_2_O (*X* = 2, 6),^31^ MgCl_2_·*X*H_2_O (*X* = 4 and 6),^32^ MgBr_2_·6H_2_O and NaBr·2H_2_O. We note that in an earlier study carried out using same MPSC set-up, NaCl/NaBr particles were concluded to be devoid of water,^4^ although here we find that the NaBr particles are not completely dry as seen in spectrum #36. We have recently studied the hydration state of CaCl_2_ particles also with XAS, using the same MPSC set-up and by varying the drying efficiency.^9^ The O 1s edge resembled “liquid-like” characteristics, but was strongly distorted by the salt which implies on high salt concentration. The spectra indicated that water molecules were present in the first coordination shell of Ca^2 +^ and Cl^−^ ions, but no clear dependence on RH was observed in the probed 16–85% RH regime suggesting that the local hydration structures remained similar. Interestingly, the spectra did not fully resemble that of an aqueous CaCl_2_ solution nor solid CaCl_2_, but rather an intermediate case. The present XPS results are in line with these observations.

### 3.4 Structural properties

**Fig. 8:**
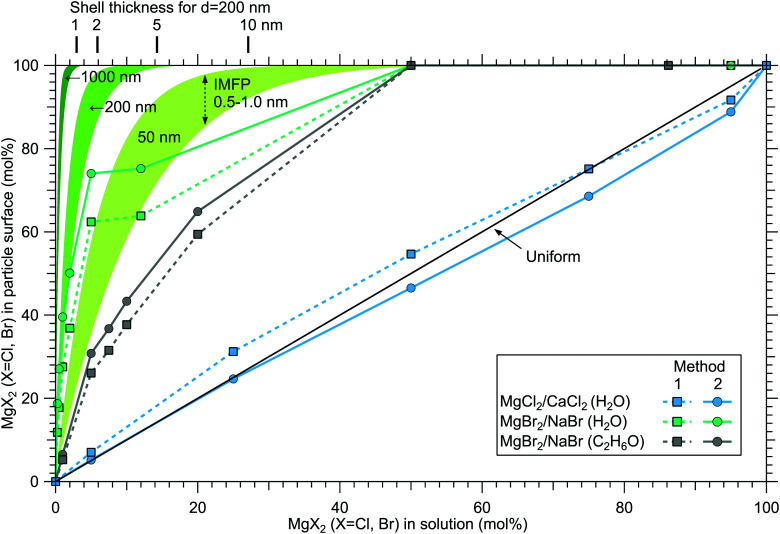
Surface concentration of MgX_2_ (X = Cl,Br) plotted as a function of the molar mixing ratio in the atomised solution, obtained as 100 at%(Mg)/(at%(Mg) + at%(Ca,Na)). For comparison, curves expected from particles with core–shell and uniform structures are shown in green (for diameters 50, 200 and 1000 nm) and black, respectively.

Although a high degree of structural variation is expected between individual particles, the results do provide some qualitative insights to the overall particle structures. Fig. 8 summarises the results of this work, showing the surface compositions of all three investigated classes of particles. For comparison, approximate curves providing a sense of scale for particles with core–shell or uniform structures are shown, based on a simple geometrical consideration on volumetric filling of the two species. A planar surface is used as a first approximation, a more detailed analysis of *e.g.* surface layer thickness would require more information of particle morphology, usually observed to be cubic for dry salt particles.^33,34^ The core–shell curves are obtained by integrating the depth decaying photoelectron intensity starting from the outermost surface. The IMFP is not exactly known, but is expected to lie in the 0.5–1.0 nm range typical for inorganic compounds.^13^ The photoelectron signal is obtained exclusively from the shell material until the shell thickness diminishes to the order of the probe depth. Note that for these curves the horizontal axis indicates the shell volume as % of the total particle volume, which is analogous to mol% only when the molar volumes of the two salts are similar. Analogously, the hydration states of the salts influence the surface enrichment factor, which along with differences in the efflorescence dynamics (and slightly different average particle size) may also partly explain the observed difference in surface enrichment efficiency of MgBr_2_ when either water or ethanol is used as a solvent.

The surface compositions for MgCl_2_/CaCl_2_ particles, as can be seen in Fig. 8, are very close to the uniform distribution curve (which is independent on particle size). Whether the two species are segregated or mixed throughout the particles is not apparent since both cases can similarly produce the observed spectra. The observed uniform distribution can result from cocrystallization, separate crystal domains (where slight enrichment of the minority component should occur,^6^ which is not clearly manifested in the data), or if the particle surface is not fully crystalline. The presence of both Ca^2 +^ and Mg^2 +^ with “uniform” abundances would also be expected if the particles were still in liquid state, although as discussed above, we estimate that there should not be much excess water in the particles. It is however noteworthy that both MgCl_2_ and CaCl_2_ can exist as supersaturated brines even at <10% RH.^31,32^

In MgBr_2_/NaBr particles, when the atomised solution is MgBr_2_ rich, the complete absence of Na at the surface suggests that in this range the particles dominantly exhibit core–shell type structures. This conclusion would also fit the pattern of reported core–shell configurations on other similar particles, NaCl/MgCl_2_^32^ and NaCl/CaCl_2_.^7^ In all three cases, it is the salt with divalent cations that covers the surface. When the atomised solution is NaBr-rich, the results are found to settle between the uniform and core–shell curves. This may reflect irregular or incomplete coverage of MgBr_2_ which have been observed for other structurally similar particles with imaging techniques.^3,32,35^ Alternatively, the salts could be partially mixed, although the complete absence of Na at the surface with low concentrations is more consistent with segregation.

Surface segregation has been previously discussed in terms of sequential crystallisation of the two species due to their different efflorescence points, which may well be the case here for NaBr/MgBr_2_ as well.^4,7,32^ Accordingly, the reason that in our experiment MgCl_2_/CaCl_2_ (H_2_O) particles do not manifest core–shell structures may owe to the fact that both species have comparably low efflorescence points ≪10% RH.^31,32^ Differences in solubilities have been also considered as a plausible reason for segregation.^36^ Zelenov and Aparina reported a study of surface segregation of salt films, quantified using NO_3_ uptake in a flow reactor.^37^ They observed a correlation between the surface composition and the difference in deliquescence RH (DRH) of the binary salt mixtures: for crystal hydrate salt – anhydrous salt pairs, the difference in DRH points are large, resulting in enhanced surface abundance of the hydrate. In contrast, the crystal hydrate salt – crystal hydrate salt pairs of NaI·2H_2_O/NaBr·2H_2_O and MgBr_2_·6H_2_O/MgCl_2_·6H_2_O did not show significant surface segregation even though their solubilities differ, explained by the similarity in their DRH values. In our case, MgCl_2_·6H_2_O and CaCl_2_·6H_2_O have very similar DRHs (∼28–33%)^38^ and ERHs (<10%) as mentioned earlier. Their solubilities differ slightly, being 5.9 mol kg^−1^ for MgCl_2_·6H_2_O and 7.2 mol kg^−1^ for CaCl_2_·6H_2_O.^39^ For NaBr/MgBr_2_·6H_2_O pair, the DRH of MgBr_2_·6H_2_O is ∼32%, thus much lower than that of NaBr·2H_2_O (58%).^40^ Their solubilities are 9.2 mol kg^−1^ and 5.5 mol kg^−1^ for NaBr and MgBr_2_·6H_2_O, respectively.^39^ They are both marked as soluble in ethanol in the CRC Handbook,^39^ but numerical data is not available. Thus, the differences in DRH/ERH seem to correlate better with the observed (lack of) surface segregation than solubility. However, it should be kept in mind that the efflorescence dynamics can change as a function of the salt mixing ratio,^32^ and enrichment may be different in aerosols which have excess water in them as in aqueous droplet phase properties at the liquid–vapor interface (*e.g.* ion polarisabilities) drive surface enhancement.^41^ Thus, in future, experiments carried out for particles as a function of humidity would be valuable for better understanding of the segregation dynamics.

The surface composition is further reflected in the BEs of the probed electronic levels, the exact values of which are sensitive to the local chemical environment (chemical shift). Particularly strong and gradual BE shifts are observed in the MgBr_2_/NaBr (H_2_O) particles as the surface composition changes from Mg- to Na-dominant (see Fig. 3, 7 and ESI† Fig. S1). Nearly linear shifting of Br 3d, Mg 2p and O 1s levels are observed with increasing Na concentration throughout the entire concentration range, but the O 1s and Mg 2p levels show an especially prominent shift to lower BE (by ∼0.5 eV for Mg 2p and <1 eV for O 1s) when the Mg concentration decreases to <5 mol%, (which is not observed in the Br 3d and Na 2s/2p levels). Similar shifts are not observed in the ethanol case, which implies that water plays a role in their origin. The O 1s BE in particles generated from MgBr_2_ alone is similar to that in particles containing the other dicationic salts, MgCl_2_ and CaCl_2_ (#29–31). However, a larger spread in O 1s energies is observed when both Na and Mg are abundant at the particle surface, and the BEs of these samples settle between the BEs of particles grown from pure MgBr_2_·6H_2_O and pure NaBr aqueous solutions. The total O 1s BE shift between pure bromide samples is about 2.7 eV. Reproduction of for example the 2% MgBr_2_ O 1s spectrum (#34) requires at least two Gaussian peaks, while one peak suffices for the pure MgBr_2_ and NaBr spectra. At least two peaks can be considered to arise as water molecules are associated with either Mg^2 +^ or Na^+^. Some spectra, *e.g.* #4 in Fig. 2 and #25 in Fig. 3, exhibit a small uniform shift at all the probed levels, which do not clearly correlate with changes in the particle surface composition. This is likely associated with a varying work-function like potential due to charging of the particles (time-dependent shifting of the spectra was also occasionally observed).

## 4 Conclusions

The surface compositions of non-supported submicron MgCl_2_/CaCl_2_ and MgBr_2_/NaBr particles formed from aqueous and organic solution droplets have been determined as a function of their mixing ratio in the atomised solution. In MgCl_2_/CaCl_2_ (H_2_O) particles, no surface enrichment of either of the species was observed. Both species were found in the particle–vapor interface with relative abundances reflecting their mixing ratio in the parent solution. In mixed MgBr_2_/NaBr (H_2_O or C_2_H_6_O) particles, MgBr_2_ was the dominant species found at the particle surface even with only minute concentrations in the atomised solution, indicative of core–shell type structures. The surface enrichment of MgBr_2_ was found to be more efficient in H_2_O solutions over C_2_H_6_O solutions, where differences in efflorescence dynamics and the number and structural configuration of remaining solvent molecules in the particles are likely to play a role. All of the here investigated salts were also observed to retain water in the particles even after passing through a drying stage and introduction to a high vacuum environment through an aerodynamic aerosol inlet, indicating their tendency to be hydrated at the particle–vapor interface even in low humidity environments.

The present study concerns the partitioning of the four most abundant atomic ions of seawater, Cl^−^, Na^+^, Mg^2 +^ and Ca^2 +^, in submicron particles formed from liquid droplets. The dominant inorganic compound of SSA is NaCl, but NaCl is in fact often completely absent from the particle–vapor interface and all of the here studied compounds have been observed to be surface enriched over NaCl.^3,4,7,32^ Considering that both NaCl/MgCl_2_^32^ and NaCl/CaCl_2_^7^ form core–shell type structures, the present investigation of mixed MgCl_2_/CaCl_2_ particles further implies that when both MgCl_2_ and CaCl_2_ are present (and surface enriched over NaCl), both species are likely to be found on the outermost particle surface layer. It was recently observed that NaBr is enriched over NaCl in submicron particles,^4^ which should be accounted for in considering the rate of Br-involving heterogeneous phase reactions in the atmosphere. Apparently, MgBr_2_ has an even higher surface propensity than NaBr. The results presented here emphasise the role of surface enrichment phenomena in aerosol particles, showcasing its extremes.

## Conflicts of interest

There are no conflicts to declare.

## Supplementary Material

CP-024-D1CP04953D-s001
