# Supplementary material for: Solvent and cosolute dependence of Mg surface enrichment in submicron aerosol particles
Source: Phys Chem Chem Phys. 2022 Jan 14;24(5):2934–43. doi: 10.1039/d1cp04953d (PMC8809137; doi:10.1039/d1cp04953d)
Supplement: CP-024-D1CP04953D-s001 [file CP-024-D1CP04953D-s001.pdf]

Electronic Supplementary Material (ESI) for Physical Chemistry Chemical Physics.
This journal is © the Owner Societies 2022

Supplementary information: Solvent and cosolute dependence of Mg surface enrichment in submicron aerosol particles

Eetu Pelimanni[1], Clara-Magdalena Saak[2,3], Georgia Michailoudi[1], Nønne Prisle[1,4], Marko Huttula[1], and Minna Patanen[1]

[1]Nano and Molecular Systems Research Unit, Faculty of Science, Box 3000, 90014 University of Oulu, Finland.
[2]Department for Physics and Astronomy, Uppsala University, Box 516, 75120 Uppsala, Sweden
[3]University of Vienna, Department of Physical Chemistry, Währinger Straße 42, 1090 Vienna, Austria
[4]Center for Atmospheric Research, Faculty of Information Technology and Electrical Engineering, P. O. Box 4500, 90014 University of Oulu, Finland

1 Details of "Method 2"

The number of Ca atoms per each Cl atom (X_{Ca}) at the surface of mixed $CaCl_2$/$MgCl_2$ particles from solution i is obtained by normalisation of the peak area ratio to that in pure $CaCl_2$ reference p as

$$X_{\mathrm{Ca}} = \frac{A^i_{\mathrm{Ca3p}}/A^i_{\mathrm{Cl3s}}}{A^p_{\mathrm{Ca3p}}/A^p_{\mathrm{Cl3s}}} \cdot \frac{1}{2}. \tag{1}$$

Similarly, the number of Mg atoms per each Cl atom is

$$X_{\mathrm{Mg}} = \frac{A^i_{\mathrm{Mg2p}}/A^i_{\mathrm{Cl3s}}}{A^p_{\mathrm{Mg2p}}/A^p_{\mathrm{Cl3s}}} \cdot \frac{1}{2}. \tag{2}$$

The 1/2 multiplications account for the ion stoichiometry. The at%s are then

$$\mathrm{at\%(Ca)} = 100 \cdot \frac{X_{\mathrm{Ca}}}{X_{\mathrm{Ca}} + X_{\mathrm{Mg}} + X_{\mathrm{Cl}}}, \tag{3}$$

$$\mathrm{at\%(Mg)} = 100 \cdot \frac{X_{\mathrm{Mg}}}{X_{\mathrm{Ca}} + X_{\mathrm{Mg}} + X_{\mathrm{Cl}}}, \tag{4}$$

and

$$\mathrm{at\%(Cl)} = 100\% - \mathrm{at\%(Ca)} - \mathrm{at\%(Mg)}, \tag{5}$$

where $X_{Cl} = 1$. Similarly for NaBr/$MgBr_2$ particles, one has

$$X_{\mathrm{Na}} = \frac{A^i_{\mathrm{Na2p}}/A^i_{\mathrm{Br3d}}}{A^p_{\mathrm{Na2p}}/A^p_{\mathrm{Br3d}}} \cdot \frac{1}{1} \tag{6}$$

and

$$X_{\mathrm{Mg}} = \frac{A^i_{\mathrm{Mg2p}}/A^i_{\mathrm{Br3d}}}{A^p_{\mathrm{Mg2p}}/A^p_{\mathrm{Br3d}}} \cdot \frac{1}{2}. \tag{7}$$

Then

$$\mathrm{at\%(Na)} = 100 \cdot \frac{X_{\mathrm{Na}}}{X_{\mathrm{Na}} + X_{\mathrm{Mg}} + X_{\mathrm{Br}}}, \tag{8}$$

$$\mathrm{at\%(Mg)} = 100 \cdot \frac{X_{\mathrm{Mg}}}{X_{\mathrm{Na}} + X_{\mathrm{Mg}} + X_{\mathrm{Br}}}, \tag{9}$$

$$\mathrm{at\%(Br)} = 100\% - \mathrm{at\%(Na)} - \mathrm{at\%(Mg)}. \tag{10}$$

2 Binding energies

The BEs are plotted in Fig. 1 as a function of the solute mixing ratio, obtained from single peak fits to the spectra, although it is apparent that the photoelectron peaks represent the full structural diversity at the particle surface and more than a single well-defined chemical state is expected. As noted in the main article, direct comparison of the BEs to other reference systems is not straightforward due to potential charging of the particles. We may however note that the observed BEs do not agree with those reported from aqueous solutions even if shifting of the energy scale is allowed for to account for a calibration offset. This indicates that the particle surface structure differs from that of aqueous solutions, which is in line with that the estimated water content is low.

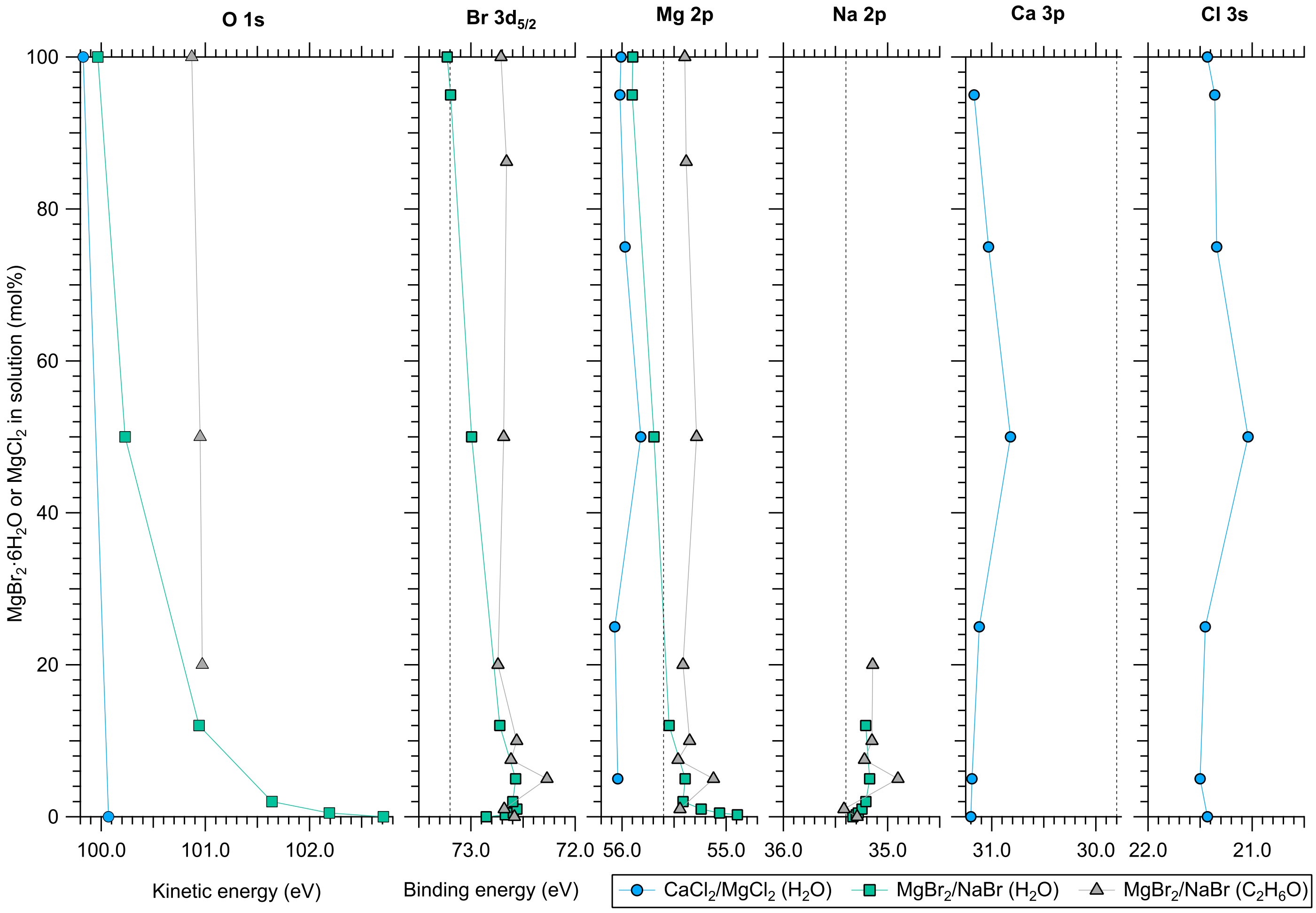

Figure 1 BEs as a function of the mixing ratio in bulk solution. For comparison, BEs of ions in aqueous solutions are indicated by the dotted lines for Ca^{2+} 3p[1], Mg^{2+} 2p[2], Na^{+} 2s[3], Na^{+} 2p[3] and Br^{-} 3d[3]. For Cl 3s a range of values has been given (see e.g. Ref. 4 and Refs. therein).

3 Details of the prepared solutions

Details of the prepared solutions are given in table 1.

Table 1 Details of the atomized solutions. Concentrations are calculated using the following molar masses: $M_{mol}(MgCl_2)$ = 95.211 g/mol, $M_{mol}(CaCl_2{\cdot}2H_2O)$ = 146.98 g/mol, $M_{mol}(MgBr_2{\cdot}6H_2O)$ = 292.204 g/mol, M_{mol}(NaBr) = 102.89 g/mol. GM = Geometric Mean size and GSD = Geometric Standard Deviation of the number concentration measurement with the SMPS.

Spec.	Solvent	Solute 1			Solute 2			Total		GM	GSD
#			mmol/L	mol%		mmol/L	mol%	g/L	mmol/L	nm	
1, 29	H_2O	$MgCl_2$	20.96	**100**				2.0	20.96		
2	H_2O	$MgCl_2$	19.13	**95**	$CaCl_2{\cdot}2H_2O$	1.01	**5**	2.0	20.15		
3	H_2O	$MgCl_2$	13.85	**75**	$CaCl_2{\cdot}2H_2O$	4.63	**25**	2.0	18.48		
4	H_2O	$MgCl_2$	8.16	**50**	$CaCl_2{\cdot}2H_2O$	8.18	**50**	2.0	16.35	180	1.8
5	H_2O	$MgCl_2$	3.73	**25**	$CaCl_2{\cdot}2H_2O$	11.18	**75**	2.0	14.91		
6	H_2O	$MgCl_2$	0.70	**5**	$CaCl_2{\cdot}2H_2O$	13.29	**95**	2.0	13.99		
7, 30	H_2O				$CaCl_2{\cdot}2H_2O$	13.76	**100**	2.0	13.76	170	1.7
8	H_2O	Pure water									
9	H_2O	$MgBr_2{\cdot}6H_2O$	17.39	**100**				5.1	17.39	170	1.7
10	H_2O	$MgBr_2{\cdot}6H_2O$	17.40	**95**	NaBr	0.95	**5**	5.2	18.35	170	1.7
11, 32	H_2O	$MgBr_2{\cdot}6H_2O$	12.68	**50**	NaBr	12.80	**50**	5.0	25.48	190	1.7
12, 33	H_2O	$MgBr_2{\cdot}6H_2O$	4.78	**12**	NaBr	35.03	**88**	5.0	39.81		
13	H_2O	$MgBr_2{\cdot}6H_2O$	2.29	**5**	NaBr	42.46	**95**	5.0	44.76	180	1.6
14, 34	H_2O	$MgBr_2{\cdot}6H_2O$	0.94	**2**	NaBr	45.93	**98**	5.0	46.87	200	1.7
15	H_2O	$MgBr_2{\cdot}6H_2O$	0.47	**1**	NaBr	45.73	**99**	4.8	46.20	170	1.7
16, 35	H_2O	$MgBr_2{\cdot}6H_2O$	0.23	**0.5**	NaBr	45.33	**99.5**	4.7	45.56	140	1.6
17	H_2O	$MgBr_2{\cdot}6H_2O$	0.12	**0.25**	NaBr	48.25	**99.75**	5.0	48.37		
18	H_2O				NaBr	30.85	**100**	3.2	30.85		
19, 37, 38	C_2H_6O	$MgBr_2{\cdot}6H_2O$	5.72	**100**				1.7	5.72	300	1.7
20	C_2H_6O	$MgBr_2{\cdot}6H_2O$	0.51	**86**	NaBr	0.08	**14**	0.2	0.59	170	1.6
21, 39	C_2H_6O	$MgBr_2{\cdot}6H_2O$	1.01	**50**	NaBr	1.03	**50**	0.4	2.04	120	1.5
22, 40	C_2H_6O	$MgBr_2{\cdot}6H_2O$	2.38	**20**	NaBr	9.49	**80**	1.7	11.86	170	1.5
23	C_2H_6O	$MgBr_2{\cdot}6H_2O$	0.36	**10**	NaBr	3.19	**90**	0.4	3.55	120	1.5
24	C_2H_6O	$MgBr_2{\cdot}6H_2O$	0.98	**7.5**	NaBr	12.20	**92.5**	1.5	13.18	150	1.5
25	C_2H_6O	$MgBr_2{\cdot}6H_2O$	0.74	**5**	NaBr	14.22	**95**	1.7	14.96		
26, 41	C_2H_6O	$MgBr_2{\cdot}6H_2O$	0.18	**1**	NaBr	15.84	**99**	1.7	16.02		
27, 42	C_2H_6O				NaBr	16.20	**100**	1.7	16.20	190	1.6
28, 43	C_2H_6O	Pure ethanol									
31	H_2O	$MgBr_2{\cdot}6H_2O$	17.32	**100**				5.1	17.32		
36	H_2O				NaBr	48.61	**100**	5.0	48.61		

References

[1] W. Pokapanich, N. V. Kryzhevoi, N. Ottosson, S. Svensson, L. S. Cederbaum, G. Öhrwall and O. Björneholm, *Journal of the American Chemical Society*, 2011, **133**, 13430–13436.

[2] G. Öhrwall, N. Ottosson, W. Pokapanich, S. Legendre, S. Svensson and O. Björneholm, *The Journal of Physical Chemistry B*, 2010, **114**, 17057–17061.

[3] B. Winter, R. Weber, I. V. Hertel, M. Faubel, P. Jungwirth, E. C. Brown and S. E. Bradforth, *Journal of the American Chemical Society*, 2005, **127**, 7203–7214.

[4] A. P. Gaiduk, M. Govoni, R. Seidel, J. H. Skone, B. Winter and G. Galli, *Journal of the American Chemical Society*, 2016, **138**, 6912–6915.
